# A Combination Operating Fracture and Orthapædic Table

**Published:** 1907-03

**Authors:** J. H. Downey

**Affiliations:** Gainesville, Ga.


### A COMBINATION OPERATING FRACTURE AND ORTHAPAEDIC TABLE.*

J. H. Downey, M. D., Gainesville, Ga.

I want to exhibit to you a combination operating, fracture and orthopaedic table, which I have been at work on for some two years. I would like to have the expression of the profession of the merits and demerits, and any friendly criticism, I assure you, will be appreciated. It is my aim to make this method and apparatus indispensable to the Surgeon, and, by your kind suggestion and friendly criticism, will I be able to do it?

I will first show you extension in the straight direction, and how I would apply a plaster cast in two sections to a Pott's Fracture of the ankle First, put on a plaster slipper, allow it to set, then apply your extension and after reduction apply the upper section. (Note with what ease and certanty you make your extension and that it holds just where you get it), then one quarter section of the top drops out of the way and gives you every access to the patient.

Next is Fracture of the upper third of the tibia and fibula. Here the cast must be applied in three sections, first the slipper, then the elbow at the knee, allow them to set, fasten foot to foot-piece, throw the arch forward and make traction upward and backward, when fragments are in apposition apply middle section. In fracture of the femur the leg is placed in the same position and the cast applied in two sections, first, from base of the toes to just above the knee, foot is fastened to foot-piece, knee-strap arranged so as to grasp the head of the femur, and traction made. It makes no difference where the bone is fractured, this position and method will be found the most satisfactory. It gives normal muscular relaxation and absolute fixa-

*Read before the Fulton County Medical Society Feb. 7th, 1907.

tion, the patient being able to get in and out of bed without assistance, sit in a chair, or walk, with the aid of crutches, any time after this plaster sets.

I think all will agree with me, that fractures of the clavicle is one of the hardest to get good results with our present method, and I want to go over a simple way of applying a light plaster yoke, which will hold the bones just as you place them. Patient placed on table with back of head on telescope upper part of table, spine on spinal support, sound shoulder tied to side of table, then fragment brought into apposition and injured shoulder fastened so patient can't move, then pad the chest, take a two-inch wide adhesive strip on each side, begin at trochanters, and go up the side to axilla and out two inches under the arm. Apply plaster, begin on line with the nipple and go up to axilla and a double "figure of eight" around shoulders and neck, then a back and forth application on each side of neck, then loosen strip under the arm and bring down to the side, forming a loop at the end, then apply another layer of plaster and you have absolute fixation of clavicle and both shoulders. (A window can be cut in plaster for inspection when desired.)

To apply plaster jacket, remove finger from center post and set in its place the shepherd crook telescope, the head piece up to full extent, sit patient on table with feet through the center, slip the clamp on the upright, down across the legs, apply neck strap and make your extension, remove all other attachments and drop the lower part of the table and it leaves the patient with spine fully extended and with access to every part without any movement of patient or aparatus.

The table can also be used for general or special operations and a quick Trendeenburg adjustment.

